# Complications of bone-anchored prostheses for individuals with an extremity amputation: A systematic review

**DOI:** 10.1371/journal.pone.0201821

**Published:** 2018-08-09

**Authors:** Robin Atallah, Ruud A. Leijendekkers, Thomas J. Hoogeboom, Jan Paul Frölke

**Affiliations:** 1 Department of Orthopaedics, Radboud university medical center, Nijmegen, the Netherlands; 2 Department of Orthopaedics, Physical Therapy, Radboud university medical center, Nijmegen, the Netherlands; 3 Radboud Institute for Health Sciences, IQ Healthcare, Radboud university medical center, Nijmegen, the Netherlands; 4 Department of Surgery, Radboud university medical center, Nijmegen, the Netherlands; Indiana University School of Medicine, UNITED STATES

## Abstract

**Background:**

This study aimed to provide an overview of device-related complications occurring in individuals with an upper or lower extremity amputation treated with a screw, press-fit or other type of bone-anchored implant as well as interventions related to these complications.

**Method:**

A systematic literature search was conducted in the MEDLINE, Cochrane, EMBASE, CINAHL and Web of Science databases. The included studies reported on device-related complications and interventions occurring in individuals with bone-anchored prostheses. The outcomes evaluated were death, infection, bone/device breakage, implant loosening, soft tissue complications, systemic events, antibiotic and surgical treatment. Subgroup analyses were performed for the following groups: a) implant type (screw, press-fit and other types of implants) and b) level of amputation (transfemoral, transtibial and upper extremity amputation).

**Results:**

Of 309 studies, 12 cohort studies were eligible for inclusion, all of which had methodological shortcomings and 12 studies were excluded due to complete overlap of patient data. Implant infection were rare in certain transfemoral implants (screw: 2–11%, press-fit: 0–3%, Compress: 0%) but common in transtibial implants (29%). The same was observed for implant loosening, in transfemoral (screw: 6%, press-fit: 0–3%, Compress: 0%), transtibial implants (29%) as well as for upper extremity implants (13–23%). Intramedullary device breakage were rare in transfemoral implants (screw: 0%, press-fit: 1%, Compress: unknown) but frequent in individuals with transradial implants (27%) and absent in transtibial implants. Soft tissue infections and complications were common and underreported in most articles.

**Conclusions:**

Major complications (e.g. implant infection, implant loosening and intramedullary device breakage) are rare in transfemoral bone-anchored prosthesis and seem to occur less frequently in individuals with press-fit implants. Minor complications, such as soft tissue infections and complications, are common but are substantially influenced by the learning curve, implant design and surgical technique. Data for patients treated with a transtibial, upper extremity or Compress implant are underreported, precluding definitive conclusions. There is a need for either an international database to report on or a standard core set of complications as well as the need to follow classification systems that result in unequivocal data.

## Introduction

The prevalence of individuals with extremity amputation is high and is only expected to increase in the coming years.[1, 2] Large differences occur among different parts of the developed world depending largely on the prevalence of peripheral vascular disease, diabetes and combat-related activities.[3] Most lower limb amputations are due to vascular disease, with the incidence increasing annually, while upper limb amputation is most often the result of trauma.[1, 2]

For the past six centuries, the rehabilitation of individuals with an upper or lower extremity amputation has been achieved with socket-mounted prostheses.[4] Despite significant technological innovations to socket materials, liners and design,[5] individuals with an upper or lower extremity amputation still exhibit significant socket-residuum interface problems, such as skin irritation, pain and problems with prosthetic fixation.[6–10]

Approximately 56% of individuals with an upper and 80–95% with a lower extremity amputation use a prosthetic limb, with a rate of dissatisfaction with the prosthesis ranging from 18–57%.[11–14] Skin problems are frequent in both upper and lower prosthetic limb users, ranging from 34–63% of all users [8, 15–21], and falling occurs in roughly half of individuals with a lower limb amputation due to poor proprioception and disbalance.[7, 22] Problems with prosthetic fixation and weight are more prevalent in individuals with upper extremity amputation.[10, 12] These socket-residuum interface problems lead to prosthesis intolerance and abandonment and have a severe impact on people’s activity levels and quality of life.[6, 9, 16, 23–25]

The only way to eliminate the socket-residuum interface and prevent the occurrence of these problems is by directly attaching the prosthesis to the bone of the residual limb via the process of osseointegration, which is defined as the direct connection of a ‘nonvital’ component incorporated in living bone.[26] This technique, originating from the field of dentistry in 1965, has been well established for the treatment of the edentulous jaw for many years, demonstrating a 5 and 10-year survival of dental implants in mandibular bone of 98% and 95%, respectively.[27–29] Bone-anchored hearing aids have been developed using this technique and have been applied on a world–wide scale since 1977, with 5-year implant survival rates of 90–95%.[30] Since its first introduction in 1990 in individuals with amputation, bone-anchored prostheses offer multiple potential benefits for the treatment of selected individuals with amputations experiencing socket-related problems. These potential benefits include improved osseoperception, prosthesis wearing time, a larger hip range of motion, and reduced oxygen consumption while walking,[31–36] which are associated with an improved mobility level, walking ability and overall quality of life.[32, 34, 37, 38] Since 1990,[26] bone-anchored prostheses have been used predominantly in individuals with a non-vascular cause of amputation, but small series have already been published showing the results of osseointegration treatment in individuals with stable vascular disease.[39, 40]

Several certified bone-anchored implants are currently available for humans: the Osseointegrated Prosthesis for the Rehabilitation of Amputees (OPRA),[32, 41–43] which is a screw implant made of titanium alloy. Also currently available are the Integral Leg Prosthesis (ILP, previously known as Endo-Exo Femur/Tibia Prosthesis; EEFP/EETP)[34, 44–50] and the Osseointegration Group of Australia-Osseointegration Prosthetic Limb (OGAP-OPL);[43], which are both press-fit implants, made of cobalt-chromium-molybdenum or titanium alloy respectively. Several newer systems are currently under development of which some have reached the stage of clinical experiments in humans.[51, 52] Initially, bone-anchored prostheses have been implanted in a two-stage procedure similar to their dental pre-ancestors, with an interval of six months and six to eight weeks for the screw and press-fit implants, respectively.[41, 43, 46] A protocol for single stage implantation of an osseointegrated prosthesis has recently been published, for which results regarding safety and efficacy remain to be evaluated.[53]

Over the last few years, multiple clinical studies have been performed to evaluate complications and the survival of bone-anchored prostheses for the treatment of individuals with upper and lower extremity amputation. At present, no systematic evaluation of complications after upper extremity amputation has been published. Reviews by van Eck et al.[54], Hebert et al.[55] and Al Muderis et al.[56] evaluated the complication rate in individuals restricted to lower extremity bone-anchored prosthesis. However, none of these reviews stratified the complication rate at the amputation level. Furthermore, van Eck et al. and Al Muderis et al. did not stratify for the type of bone-anchored prosthesis, resulting in limited clinical usability. The latter is important because the fixation principle of these implants are different because they are being developed for dentistry (screw) and orthopedic surgery (press-fit).[57, 58] Another limitation was that insight in the level of overlap in participants in the included studies was not [54, 56] or insufficiently provided [55] despite the often partial and occasionally even total overlap of the embedded cohort of participants.

Therefore, the two aims of this study were to provide (a) a stratified overview of device-related complications in individuals with a lower or upper extremity amputation treated with a screw, press-fit or other type of bone-anchored prostheses and (b) a stratified overview of the complication-related interventions that occur in these individuals treated with bone-anchored prosthetics.

## Methods

### Design

This systematic review of published, peer-reviewed articles with original data was conducted following the guidelines of the PRISMA statement.[59] The initial review protocol has been registered in the PROSPERO database.[60] The focus of the initial review protocol was screw or press-fit bone-anchored prostheses, nonetheless upon writing we decided to include other types of bone-anchored prostheses following the classification by Thesleff et al. [52]

### Data collection

A comprehensive search was performed by the second author (RL) on 8 January 2018 in MEDLINE (accessed via PubMed), Cochrane Central Register of Controlled Trials, Embase (accessed via OvidSP), CINAHL, Web of Science and System for information on Grey Literature. Several combinations of terms and expressions were used, including both MeSH and free text terms. The final search string included (osseointegrat* OR osseo-integrat* OR bone-anchored prosthe*) AND (amput*). No date limits or geographical restrictions were used. Search strings for each database are provided in S1 Appendix.

### Eligibility criteria

The eligibility of studies was independently assessed by RA and RL. We included articles of randomized controlled trials, controlled clinical trials and prospective and retrospective observational studies (including before-after, cohort and case–control studies). Articles were included if they reported device-related complications and/or complications related to interventions in people with an upper and/or lower extremity amputation treated with bone-anchored prostheses. We excluded studies that were not in the English, Dutch or German language. Furthermore, we excluded studies that presented completely duplicated data, studies that presented no original data (e.g., systematic reviews) and studies without having a full text. The individual studies embedded in systematic reviews were screened using the same eligibility criteria.

### Study selection

**Fig 1:**
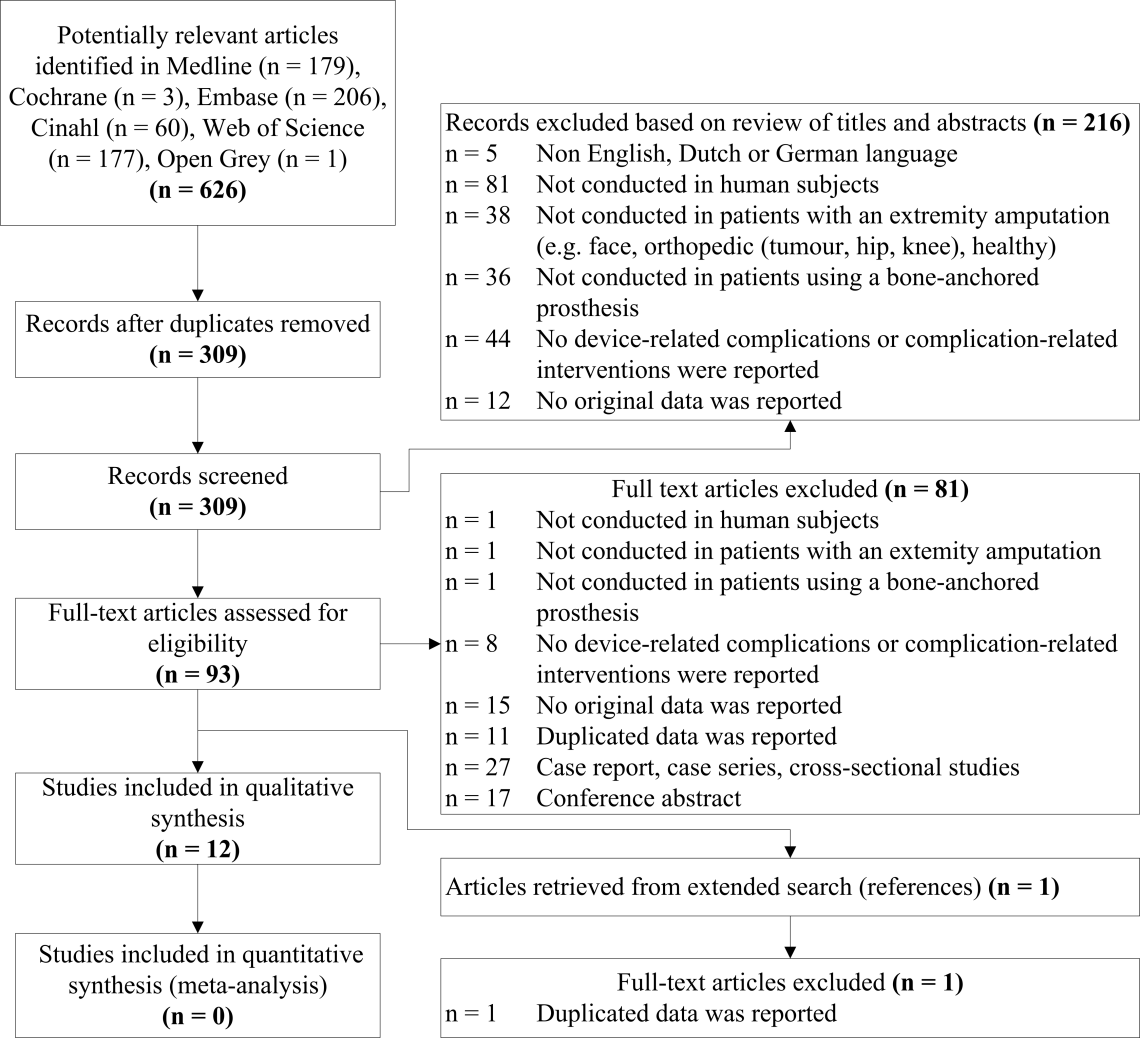
Flowchart for included studies.

Study selection was completed in two phases by two reviewers (RA, RL) independently. During the first phase, titles and abstracts of studies retrieved using the search strategy were screened to identify studies potentially meeting the inclusion criteria. The full text of these potentially eligible studies were retrieved and independently assessed for eligibility by both reviewers during the second phase. Additionally, a manual search of the reference list of the included articles was performed (Fig 1). In case of disagreement in any screening stage, conflicts were resolved in a consensus meeting. Reasons for exclusion of the title and abstract of the reviewed articles are outlined in S2 Appendix. If articles presented a partial overlapping cohort of participants, the authors were contacted to provide source data aiming to include only unique cohorts of participants. If no response was obtained after one reminder, we included all involved articles to avoid the loss of relevant data. If the cohorts of participants completely overlapped, the study with the largest cohort was included.

### Data extraction and analysis

Data extraction was performed by two authors independently (RA and RL). Again, if any discrepancies occurred, a consensus was provided in discussion. Data were extracted using a standardized form and included authors, publication year, study location, follow-up period, study design, time interval of inclusion, participant demographics, type of intervention (single or two stage surgery), type of implant (screw, press-fit or other), device-related complications (death, infection, bone fracture, device breakage, implant loosening, stoma hypergranulation, stoma redundant tissue and systemic events) and complication-related interventions (antibiotic use and surgical treatment). If possible, the level of infection was categorized using a classification system for infection based on clinical and radiographic signs, which was published by Al Muderis et al. Table 1.[61] If an article only described specific complications, all other complications were scored as “unknown”. Complications were scored as a percentage of the total individuals in which they occurred. If enough unique homogeneous studies were included with overlapping follow-up time points, a meta-analysis was conducted to pool the incidence of device-related complications and complication-related interventions. Outcomes were analyzed separately for short-term (less or equal than one-year), mid-term (two to five year) and long-term (equal or more than five-year) follow-up. If the necessary data were available, subgroup analyses were performed for the following groups: a) implant type (screw, press-fit or other) and b) level of amputation (transfemoral, transtibial and upper extremity amputation).

**Table 1:** Classification of infection.

Level of Severity	Symptoms and Signs	Treatment	Grade
Low-grade soft tissue infection	Cellulitis with signs of inflammation (redness, swelling, warmth, stinging pain, pain that increases on loading, tense)	-Oral Antibiotics	1A
		-Parenteral Antibiotics	1B
		-Surgical Intervention	1C
High-grade soft tissue infection	Pus collection, purulent discharge, raised level of C-reactive protein	-Oral Antibiotics	2A
		-Parenteral Antibiotics	2B
		-Surgical Intervention	2C
Bone infection	Radiographic evidence of osteitis (periosteal bone reaction), radiographic evidence of osteomyelitis (sequestrum and involucrum)	-Oral Antibiotics	3A
		-Parenteral Antibiotics	3B
		-Surgical Intervention	3C
Implant failure	Radiographic evidence of loosening	-Parenteral antibiotics, explantation	4

### Methodological quality

The methodological quality of the included articles was independently assessed by two reviewers (RA and RL), after which disagreements were discussed in consensus meetings. In the case of persistent disagreement, a third reviewer was consulted to mediate (TH). The methodological quality (risk of bias) was scored using the Effective Public Health Practice Project (EPHPP) Quality Assessment Tool for Quantitative Studies.[62, 63] The EPHPP was chosen because we anticipated retrieving different types of non-randomized observational studies. The EPHPP Quality Assessment Tool assesses six aspects of methodology: (1) selection bias, (2) study design, (3) control of confounders, (4) blinding of participants and investigators, (5) data collection tool validity and reliability, and (6) proportion of withdrawals and drop-outs. Every study was assessed using the tool, and the studies were rated as “strong”, “moderate” or “weak” with respect to the above-mentioned aspects using standard criteria. [62, 63] Combining the ratings of all six aspects of methodology resulted in an overall rating of quality (global rating), with studies classified as having “strong” methodology when no aspects were rated weak, “moderate” when only one aspect was rated weak and “weak” when multiple aspects of methodology were rated weak.[62, 63] Inter-rater agreement on aspects of methodology was measured with a linear, weighted Cohen’s Ƙ coefficient.[64] Values were classified as follows: 0.41–0.60: fair agreement; 0.61–0.80: good agreement; 0.81–0.92: very good agreement; 0.93–1.00: excellent agreement.[65]

## Results

### Selected studies

**Fig 2:**
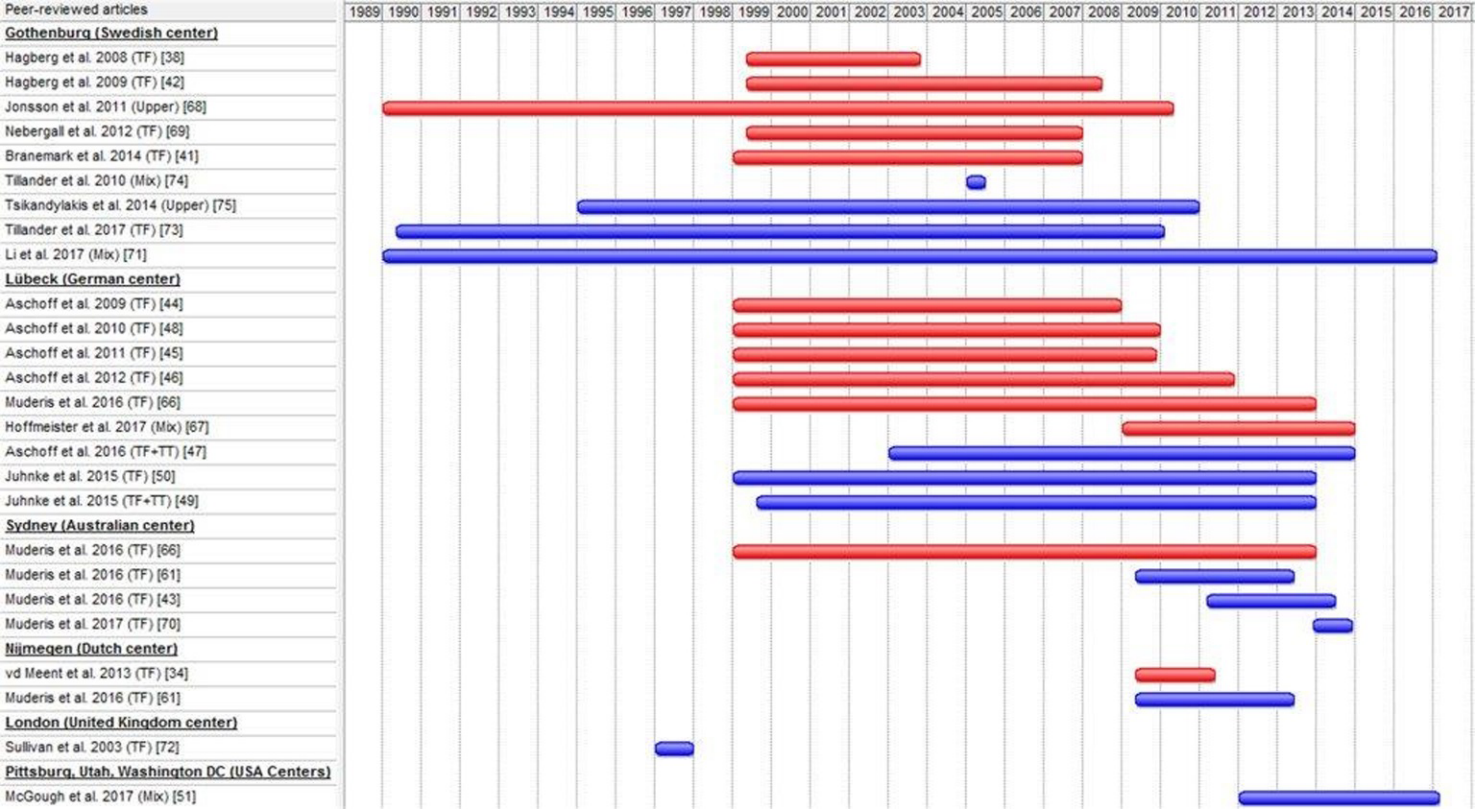
Gantt chart of overlapping data.

We identified 309 unique articles in the search and 1 from screening references (Fig 1). Twenty-four articles met our in-and exclusion criteria of which 12 articles were excluded because the cohorts of participants overlapped completely.[34, 38, 41, 42, 44–46, 48, 66–69] The 12 remaining eligible articles [43, 47, 49–51, 61, 70–75] described a total of 537 individuals with a lower and 67 individuals with an upper limb amputation. All individuals were treated with bone-anchored prostheses in eight different centers worldwide, but some articles presented partial overlapping cohorts of participants. The three articles of the Australian center had overlapping data in the period from 2011–2013 and 2013–2014,[43, 61, 70] the articles of the German center had an overlap in data in the period from 2003–2013,[47, 49, 50] the articles of the Swedish center regarding individuals with upper extremity amputation had an overlap in the period 1995–2010 [71, 75] and the article by Tillander et al. from 2010 had an unclear interval of inclusion.[74] A Gantt chart was made to provide a better overview of the amount of overlap in data between studies (Fig 2). Due to Tillander et al. [73] reporting on all the individuals with transfemoral amputation which were also partly reported on by Li et al. [71] we only included the individuals with an upper extremity amputation from the article by Li et al.

### Study characteristics

Table 2 provides the characteristics of the included articles. The 12 articles presented six retrospective cohort studies,[47, 49, 50, 70, 73, 75] three prospective cohort studies [43, 61, 74] and three cohort studies with an undefined design.[51, 71, 72] Three articles described two separate patient cohorts based on the amputation level or implant type.[47, 49, 50] We stratified our results by the number of cohorts described, resulting in a total of 15 cohorts. One of these cohorts was described by Tillander et al.[74], who used a combination of individuals with lower and upper extremity amputation and thus the outcome of this cohort will be mentioned separately to avoid clouding the overall results. The follow-up period of all cohorts ranged from 1 to 288 months no study was included with a fixed follow-up. The most common cause of amputation was trauma. One article presented cohort data from two centers in different countries.[61] Surgery was performed in eight centers in six countries: Australia [43, 61, 70], Germany [47, 49, 50], the Netherlands [61], Sweden [71, 73–75], the United Kingdom [72] and the USA [51]. The OPRA screw implant was used in Sweden and the United Kingdom, the ILP/OPL press-fit implant was used in Australia, Germany and the Netherlands and the Compress implant was used in the USA.

**Table 2:** Study characteristics.

Authors (years) Study location	Study design	Time interval inclusion of patients	Mean follow-up (months) ± SD (median) [range]	Participants (n), Implants, Sex (M/F), Level of amputation	Cause of amputation (%)	Mean age (years) at surgery ±SD (median) [range]	Mean time (years) from primary amputation to surgery SD (median) [range]	Type of Intervention: type of implant / type of alloy / type of surgery(1-step, 2-step)
Al Muderis et al., Australia (2017) [70]	Retrospective cohort	December 2013 to November 2014	14 ± ? (?) [10–30]	N = 22 (22 implants), (16 M, 6 F), 22 uni-TF	Trauma (73), Tumour (18), Infection (9)	46 ± ? (?) [20–67]	? ± ? (?) [?]	Press-fit: OPL / (titanium / 1-step
Li et al., Sweden (2017) [71]	Cohort	TR: 1990 to 2017, Thumb: 1990 to 2014, TH: 1995 to 2010	TR ? ± ? (?) [?], Thumb ? ± ? (?) [?], TH ? ± ? (96) [24–228]	N = 42 (43 implants)*, (TR: 10 M, 1 F; TH: 10 M, 3 F; Thumb: ?), 10 uni TR, 1 bi-TR, 13 uni-thumb, 18 uni-TH	TR: ?, Thumb: Trauma (85), Tumour (15), TH: ?	? ± ? (?) [?]	? ± ? (?) [?]	Screw: OPRA / Titanium / 2-step
McGough et al., USA (2017) [51]	Cohort	2012 to 2017	? ± ? (?) [?]	N = 11 (11 implants), (10 M, 1 F), 10 uni-TF, 1 uni-TH	Trauma (55), Tumour (36), Infection (9)	47 ± ? (?) [26–68]	? ± ? (?) [?]	Compress / ?? / 1-step (n = 6) and 2-step (n = 5)
Tillander et al., Sweden (2017) [73]	Retrospective cohort	May 1990 to January 2010	95 ± ? (74) [18–235]	N = 96 (102 implants), (60 M, 36 F), 90 uni-TF, 6 bi-TF	Trauma (71), Tumour (20), Ischemia (5), Infection (5), Other (1)	43 ± ? (?) [19–65]	11.5 ± ? (?) [<1–44]	Screw: OPRA / Titanium / 2-step
Al Muderis et al., Australia, The Netherlands (2016) [61]	Prospective cohort	May 2009 to May 2013	? ± ? (34) [range 24–71]	N = 86 (91 implants), (65 M, 21 F), 76 uni- TF, 5 bi-TF	Trauma (76), Tumour (13), Infection (9), Congenital (1), Other (1)	48 ± 14 (?) [25–81]	16 ± 14 (?) [?]	Press-fit: ILP/Cobalt-chromium-molybdenum/2-step
Al Muderis et al., Australia (2016) [43]	Prospective cohort	March 2011 to June 2014	22 ± ? (?) [range 1-?]	N = 50 (50 implants), (34 M, 16 F), 50 uni-TF	Trauma (64), Tumour (16), Infection (10), Congenital (4), Blast injury (6)	48 ± ? (?) [24–73]	? ± ? (?) [2–65]	Press-fit: ILP (Cobalt-chromium-molybdenum)/OPL (titanium)/2-step
Aschoff et al., Germany (2016) [47]	Retrospective cohort	January 2003 to December 2014	? ± ? (?) [?]	N = 86 (94 implants), (68 M, 18 F), 73 uni-TF, 6 bi-TF, 5 uni-TT, 2 bi-TT	Trauma (77), Tumour (8), Other (15)	[17–76]	? ± ? (?) [?]	Press-fit: EEFP + EETP = ILP = cobalt chrome molybdenum/2-step
Authors (years), Study location	Study design	Time interval inclusion of patients	Mean follow-up (months) ± SD (median) [range]	Participants (n), Implants, Sex (M/F), Level of amputation	Cause of amputation (%)	Mean age (years) at surgery ±SD (median) [range]	Mean time (years) from primary amputation to surgery SD (median) [range]	Type of Intervention: type of implant / type of alloy / type of surgery(1-step, 2-step)
Juhnke et al., Germany (cohort 1) (2015) [50]	Retrospective cohort	January 1999 to December 2008	74 ± 31 [6–144]	N = 30 (31 implants), (25 M, 5 F), 29 uni-TF, 1 bi-TF	Trauma (77), Tumour (17), Infection (3), Other (3)	46 ± 13 [17–69]	11 ± ? (?) [?]	Press-fit: ILP design A and B/Cobalt-chromium-molybdenum/2-step
Juhnke et al., Germany (cohort 2), (2015) [50]	Retrospective cohort	January 2009 to December 2013	32 ± 18 (?) [1–59]	N = 39 (42 implants), (31 M, 8 F), 36 uni-TF, 3 bi-TF	Trauma (72), Tumour (5), Infection (5), Burn (3), Other (15)	45 ± 12 (?) [24–76]	11 ± ? (?) [?]	Press-fit: ILP design C/Cobalt-chromium-molybdenum/2-step
Juhnke et al., Germany (2015) [49]	Retrospective cohort study	August 1999 to December 2013	? ± ? (?) [?]	N = 74 (80 implants), (59 M, 15 F), 63 uni-TF, 4 bi-TF, 5 uni-TT, 2 bi-TT	Trauma (76), Tumour (9), Other (15)	46 ± ? (?) [17–76]	11 ± ? (?) [?]	Press-fit: EEFP and EETP = ILP/?/2-step
Tsikandylakis et al., Sweden (2014) [75]	Retrospective cohort	1995 to 2010	? ± ? (96) [24–288]	N = 18 (18 implants), (16 M, 2 F), 18 uni-TH	Trauma (89), Tumour (11)	42 ± ? (?) [19–69]	9 ± ? (?) [2–33]	Screw: OPRA/Titanium/2-step
Tillander et al., Sweden (2010) [74]	Prospective cohort	January 2005 to June 2005	36 ± ? (?) [?] / Time BAP to inclusion: 54 ± ? (?) [3–132]	N = 39 (45 implants), (21 M, 18 F), 31 uni-TF, 1 bi-TF, 2 uni-TR, 1 bi-TR, 3 uni-TH, 1 uni-TT	Trauma (?), Tumour (?)	49 ± ? (?) [28–74]	? ± ? (?) [?]	Screw: OPRA/Titanium/2-step
Sullivan et al., United kingdom (2003) [72]	Cohort	1997	? ± ? (?) [?]	N = 11 (11 implants), (? M, ? F), 11 TF	?	? ± ? (?) [?-?}	? ± ? (?) [?-?}	Screw: OPRA/Titanium/2-step

SD = standard deviation, M = Male, F = Female, OPRA = Osseointegrated Prosthesis for the Rehabilitation of Amputees, OGAAP = The Osseointegration Group of Australia Accelerated Protocol, TF = Transfemoral, TT = Transtibial, TH = Transhumeral, TR = Transradial, Uni- = Unilateral, Bi- = Bilateral, ILP = Integral Leg Prosthesis, OPL: Osseointegration prosthetic limb (OGAP-OPL), EEFP = Endo-exo Femur Prosthesis, EETP = Endo-exo Tibia Prosthesis, BAP = Bone-anchored prosthesis, ? = Unknown/unclear.

* With exclusion of individuals with TF amputation due to the overlap with Tillander et al.[73].

Of the 604 individuals in the 15 included cohorts, 206 were treated with a screw implant, 387 were treated with a press-fit implant and 11 were treated with the Compress implant. A total of 522 individuals were treated with a transfemoral amputation (screw: 139, press-fit: 373, Compress: 10), 15 with a transtibial amputation (screw: 1, press-fit 14) and 67 individuals with an upper extremity amputation (screw: 66, press-fit: 0, Compress: 1), of which 40 had a transhumeral amputation (screw: 39, Compress: 1), 14 a transradial amputation and 13 a thumb amputation.

The mean age at the time of implantation surgery was 45, 47 and 48 years in individuals treated with a screw, press-fit or Compress implant respectively. The mean time from primary amputation to implantation was 10.3 and 12.3 years for individuals treated with a screw and press-fit implant, respectively and was not described in the article regarding the Compress implant.

In each article if possible, loss to follow-up was determined by calculating the amount of individuals lost to follow-up that were not subdivided in any other category of complications.

### Methodological quality assessment

The inter-rater agreement of the assessment expressed as ƙ was 0.93±0.04, with 96% inter-rater agreement between the two reviewers on the ratings of the individual domains of methodological quality. The most common shortcomings of the studies were failure to blind assessors and participants, lack of adjustment for confounding variables and limited validity or reliability of the data collection methods. The few disagreements about domain errors were due to errors in comprehension or differences in interpretation of the methodological quality criteria. Disagreements were resolved in a consensus meeting. Scores for the six domains of methodological quality and the global EPHPP scores are presented in table 3.

**Table 3:** Methodological quality assessment ratings based on the Effective Public Health Practice Project tool for quantitative studies.

Authors (year)	Selection bias	Study design	Confounders	Blinding	Data collection	Withdrawals and drop-outs	Global rating
Al Muderis et al. (2017)[70]	Moderate	Moderate	Weak	Weak	Weak	Strong	Weak
Li et al. (2017)[71]	Moderate	Moderate	Weak	Weak	Weak	Weak	Weak
McGough et al. (2017)[51]	Moderate	Moderate	Weak	Weak	Weak	Weak	Weak
Tillander et al. (2017)[73]	Moderate	Moderate	Weak	Weak	Weak	Strong	Weak
Al Muderis et al. (2016)[61]	Moderate	Moderate	Weak	Weak	Weak	Strong	Weak
Al Muderis et al. (2016)[43]	Moderate	Moderate	Weak	Weak	Weak	Strong	Weak
Aschoff et al. (2016)[47]	Moderate	Moderate	Weak	Weak	Weak	Weak	Weak
Juhnke et al. (2015)[50]	Moderate	Moderate	Weak	Weak	Weak	Weak	Weak
Juhnke et al. (2015[49])	Moderate	Moderate	Weak	Weak	Weak	Weak	Weak
Tsikandylakis et al. (2014)[75]	Moderate	Moderate	Weak	Weak	Weak	Weak	Weak
Tillander et al. (2010)[74]	Weak	Moderate	Weak	Weak	Weak	Strong	Weak
Sullivan et al. (2003)[72]	Moderate	Moderate	Weak	Weak	Weak	Weak	Weak

### Synthesis of results/meta-analysis

Because many cohorts partially overlapped, we could not conduct a meta-analysis. None of the contacted authors were able to provide source data. Due to the heterogeneity in follow-up time-points, we could not stratify the outcomes in short-, mid- and long-term outcomes. We stratified the outcomes of individual studies into two categories: a) implant type (screw, press-fit and other) and b) level of amputation (transfemoral, transtibial and upper extremity amputation).

### Results of individual studies

Table 4 presents the device-related complications, and table 5 presents the complication-related interventions occurring in individuals with bone-anchored prostheses.

**Table 4:** Device-related complications.

Autdors (years)	Implant type/ Level of amputation	Loss to follow-up (%) (reason)	Un-eventful course (%)	Infection (%) (grade (% of patients))	Bone fracture (%)	Device breakage, (intr., DCA/abut. (% of total))	Implant loosening (%)	Stoma hyper-granulation (%)	Stoma redundant tissue (%)	Otder soft tissue complications (%)	Systemic events (MI/PE) (%)
**Transfemoral**											
Tillander et al. (2017) [73]	Screw	2 (50 DU death, 50 ?)	?	? (grade 1–2: ?, grade 3: 13, grade 4: 11)	?	?	?	?	?	?	?
Sullivan et al. (2003) [72]	Screw	?	?	?	?	45 (abut.: 100)	?	?	?	?	?
Al Muderis et al. (2017) [70]	Press-fit	5 (DU death)	?	57 (grade 1: 48, grade 2: 10)	0	0	0	?	?	?	?
Al Muderis et al, (2016) [61]	Press-fit	?	36	34 (grade 1: 29, grade 2: 5)	3	31 (Intr.: 6 (2% of patients), DCA: 94 (29% of patients)	1	20	16	?	?
Al Muderis et al. (2016) [43]	Press-fit	6 (100 DU death)	32	42 (?)	8	2 (intr.: 100)	2	?	14	?	?
Aschoff et al. (2016) [47]	Press-fit	?	?	?	8	1 (Intr.: 100)	0	?	?	?	?
Juhnke et al. (cohort 1) (2015) [50]	Press-fit	?	20	77 (grade 1–2: ?, grade 3: ?, grade 4:3)	10	3 (?)	?	?	?	?	?
Juhnke et al. (cohort 2) (2015) [50]	Press-fit	?	87	0 (-)	5	0	3	3	3	3 (fistula)	?
Juhnke et al. (2015) [49]	Press-fit	?	?	? (grade 1–2: ?, grade 3: ?, grade 4: 3%)	10	?	?	?	?	?	?
McGough et al. (2017) [51]	Compress	?	73	0	18	?	0	?	9	?	?
Authors (years)	Implant type/ Level of amputation	Loss to follow-up (%) (reason)	Un-eventful course (%)	Infection (%) (grade (% of patients))	Bone fracture (%)	Device breakage, (intr., DCA/abut. (% of total))	Implant loosening (%)	Stoma hyper-granulation (%)	Stoma redundant tissue (%)	Other soft tissue complications (%)	Systemic events (MI/PE) (%)
**Transtibial**											
Aschoff et al. (2016) [47]	Press-fit	?	?	?	?	?	29	?	?	?	?
Juhnke et al. (2015) [49]	Press-fit	?	?	? (grade 1–2: ?, grade 3: ?, grade 4: 29%)	?	?	?	?	?	?	?
**Upper extremity**											
Li et al., (2017) [71]	Screw (Mixed upper extremity)	TR: ?, Thumb: ?, TH: 11 (at 2 year FU)	TR: ?, Thumb: 46, TH: ?	?	?	TR: 27 (Intr. 100), Thumb: ?, TH: ?	TR: ?, Thumb: 23, TH: 13	?	?	?	?
Tsikandylakis et al. (2014) [75]	Screw	0	?	44 (grade 1: 28, grade 3: 6, grade 4: 11)	0	?	?	44	?	?	?
Tillander et al. (2010) [74]	Screw (Mixed upper/lower extremity)	5 (100 unspecified NM)	?	Inclusion: 23 (grade 1–2:18, grade 3:5), FU: 49 (grade 1–2: 30, grade 3:11, grade 4: 8)	?	?	3 (100: TF)	?	?	?	?

Grading of infection = Grade 1: Superficial soft tissue, Grade 2: Deep soft tissue, Grade 3: Bone infection, Grade 4: Implant infection. DCA = Dual cone adaptor, Intr. = Intramedullary device, Abut. = Prosthetic abutment, MI = Myocardial infarction, PE = Pulmonary embolism, TF = Transfemoral, TT = Transtibial, TH = Transhumeral, DU = Device-unrelated, DR = Device-related, Loose. = Implant loosening, No-S2 = Not yet after Surgery 2, NM = Non-medical, FU: follow-up

**Table 5:** Complication-related interventions.

Authors (years)	Implant type/Level of amputation	Oral antibiotics (%)	Parenteral antibiotics (%)	Surgical debridement (%) (% of total: infection, hypergranulation, stoma redundant tissue, other)	Explantation (%) (% of total: infection, loosening, bone/implant fracture, other)	Successful re-implantation (% of explantation)	Fracture treatment (% of fractures) (conservative/surgical)
**Transfemoral**							
Tillander et al. (2017) [73]	Screw	?	?	?	17 (63: inf., 38: ?)	6 (100% of infection explantations) *	?
Sullivan et al. (2003) [72]	Screw	?	?	?	18 (100: inf.)	?	?
Al Muderis et al. (2017) [70]	Press-fit	48 (all grade 1 cases)	10 (all grade 2 cases)	29 (100: redund.)	0	NA	NA
Al Muderis et al. (2016) [61]	Press-fit	27	1	22 (27: inf., 73: redund.)	3 (33: loose., 67: intr. device break.)	100	100: surgical
Al Muderis et al. (2016) [43]	Press-fit	26	10	20 (30:inf., 70: redund.)	4 (50: loose., 50: intr. device break.)	100	100: surgical
Aschoff et al. (2016) [47]	Press-fit	?	?	? (6 of total TF+TT (100: redund.))	6 (80: inf., 20: device break.)	Unknown (38% of total explantations TF+TT)	100: surgical
Juhnke et al. (cohort 1) (2015) [50]	Press-fit	?	?	77 (100:inf.)	13 (100: inf.)	50	100: surgical
Juhnke et al. (cohort 2) (2015) [50]	Press-fit	?	?	8 (33: hyperg., 33: redund., 33: ilizarov treatment)	3 (100: loose.)	100	100: surgical
Juhnke et al. (2015) [49]	Press-fit	?	?	?	6 (unknown)	50	100: surgical
McGough et al. (2017) [51]	Compress	?	?	9 (100: redund.)	9 (100: bone fract.)	100	50: surgical in combination with implant revision, 50: awaiting revision
Authors (years)	Implant type/Level of amputation	Oral antibiotics (%)	Parenteral antibiotics (%)	Surgical debridement (%) (% of total: infection, hypergranulation, stoma redundant tissue, other)	Explantation (%) (% of total: infection, loosening, bone/implant fracture, other)	Successful re-implantation (% of explantation)	Fracture treatment (% of fractures) (conservative/surgical)
**Transtibial**							
Aschoff et al. (2016) [47]	Press-fit	?	?	? (6 of total TF+TT (100: redund.))	43 (33: inf., 67: loose.)	Unknown (38% of total explantations TF+TT)	?
Juhnke et al. (2015) [49]	Press-fit	?	?	?	57 (unknown)	25	?
**Upper extremity**							
Li et al. (2017) [71]	Screw (Mixed upper extremity)	?	?	?	TH: 19 (67: loose., 33: glenohumeral osteoarthritis) TR: ?, Thumb: ?	TH: 33	?
Tsikandylakis et al. (2014) [75]	Screw (TH)	22	?	11 (100: inf.)	17 (67: loose., 33: glenohumeral osteoarthritis)	33	?
Tillander et al. (2010) [74]	Screw (Mixed upper/lower extremity)	?	?	?	14 (60: Deep inf. (TF), 20: loose (TF), 20: Chronic skin inf. (unknown))	40	?

TF = Transfemoral, TT = Transtibial, TH = Transhumeral, Inf. = Infection, Redund. = Stoma rendundant tissue, Loose.: Implant loosening, Intr. = Intramedullary device, Fract. = Fracture, Break.: Breakage, Hypergr. = Hypergranulation tissue.

* No data on succesfull reimplantation of individuals with explantation with unknown reason.

#### Infection

The occurrence of infection was reported in 11 out of 15 cohorts (73%).[43, 49–51, 61, 70, 73–75] The infection rate ranged from 23–49% in individuals treated with screw implants compared with 0–77% in individuals treated with press-fit implant and 0% in individuals treated with the Compress implant. Soft tissue infections in the skin-penetrating area (Grade 1–2) occurred in 28% and 0–57% of individuals treated with screw and press-fit implants, respectively. Bone infection (Grade 3) occurred in 5–13% and 0% of individuals treated with screw and press-fit implants, respectively. Infections resulting in implant loosening (Grade 4) occurred in 8–11% and 3–29% of individuals treated with screw and press-fit implants, respectively.

Examination of infections rates in relation to amputation level revealed a rate of infection ranging from 0–77% in individuals with transfemoral amputation treated with press-fit implants and 44% in individuals with upper extremity amputation. The rate of infection in individuals with transfemoral amputation treated with screw implants or individuals with transtibial amputation was unkown. The rate of soft tissue infections (Grade 1–2) ranged from 0–57% in individuals with transfemoral amputation treated with press-fit implants and there was a rate of 28% in individuals with upper extremity amputation. There was no reported rate in individuals with transfemoral amputation treated with screw implants or individuals with transtibial amputation. Bone infection (Grade 3) occurred in 13% of individuals with transfemoral amputation treated with screw implants and 6% of individuals with upper extremity amputation. There was no reported rate in individuals with transfemoral amputation treated with press-fit implants or in individuals with transtibial amputation. Implant loosening due to infection (Grade 4) occurred in 0–11% of individuals with transfemoral amputation (screw-fit: 11%, press-fit: 0–3%), 29% of individuals with transtibial amputation and 11% of individuals with upper extremity amputation, all of which being individuals with transhumeral amputation.

The article by Juhnke et al.[50] was the only one reporting infection rates before and after adaptation of surgical technique and implant design and presented a decrease in infection rates from 77% to 0% in press-fit transfemoral implants. The article by Tillander et al.[74] was the only one to report the incidence of infection in individuals attending a scheduled or emergency visit who were surveyed at inclusion and three years later. The reported incidence of infection was 23 and 49% (among which 8% implant loosening) at inclusion and three years later, respectively, among a cohort of individuals with an upper- and lower-extremity amputation treated with screw implants.

#### Peri-prosthetic bone fracture

The incidence of peri-prosthetic bone fracture was described in nine of 15 cohorts (60%) with an incidence of 0% in individuals treated with a screw implant, 0–10% in individuals treated with a press-fit implant and 18% in individuals treated with the Compress implant.[43, 47, 49–51, 61, 70, 75] Three articles reported the cause of bone fracture which were falls in all studies.[43, 51, 61] All reported peri-prosthetic bone fractures occurred in individuals with press-fit transfemoral bone-anchored implants. No fractures occurred in individuals with upper extremity bone-anchored implants and no data reported on the incidence of fractures in individuals with transfemoral amputation treated with screw implants or individuals with transtibial bone-anchored implants.

#### Device breakage

The incidence of device breakage were mentioned in eight of 15 cohorts (53%) and subdivided in fractures of the intramedullary implant, of the abutment (screw) and of the dual cone adaptor (press-fit).[43, 47, 50, 61, 70–72] Device breakage occurred in 27–45% and 0–31% of individuals treated with screw and press-fit implants, respectively. These device breakages were of the abutment and intramedullary part in screw implants (transfemoral: 100% abutment, transradial: 100% intramedullary component) and mostly breakages of the dual cone adapter in press-fit implants (up to 94%). Device fractures were not reported in the cohort treated with the Compress implant.[51]

No intramedullary device breakages were reported in individuals with transfemoral amputation treated with screw implants, while intramedullary device breakages occurred in, on average, 1% of individuals with transfemoral amputation treated with press-fit implants. No device breakages were reported in individuals with transtibial bone-anchored prostheses. There was an incidence of intramedullary device breakage of 27% in individuals with transradial screw implants. The article by Juhnke et al.[50] did not specify the part of the device in which a breakage occurred.

#### Implant loosening

The incidence of implant loosening of the bone-anchored implants was reported in nine of the 15 cohorts (60%).[43, 47, 50, 51, 61, 70, 71, 74] It ranged from 3–23% and 0–29% in individuals treated with screw and press-fit implants, respectively. No implant loosening occurred in individuals treated with the Compress implant.

The rate of implant loosening was not described in individuals with transfemoral amputation treated with screw implants and was 0–3% in those treated with press-fit implants. Implant loosening occurred in up to 29% of individuals with transtibial amputation treated with press-fit implants and in 13% and 23% of individuals with transhumeral and thumb amputation respectively, treated with screw implants. Implant loosening was not reported in individuals with transradial amputation. All implants (3%) that presented with loosening in the cohort reported by Tillander et al.[74] were transfemoral screw implants.

#### Soft tissue complications

Soft tissue complications were subdivided into stoma hypergranulation, stoma redundant tissue and other soft tissue complications. The incidence of stoma hypergranulation and redundant tissue was reported in five of the 15 cohorts (33%) with other soft tissue complications also being reported in the cohort assessed by Juhnke et al. (Table 4).[43, 50, 51, 61, 75]

Stoma hypergranulation occurred in 44% and 3–20% of individuals treated with screw and press-fit implants, respectively, and was not reported in individuals treated with the Compress implant. Stoma redundant tissue was not reported in the cohorts of individuals treated with screw implants, but occurred in 3–16% and 9% of individuals treated with press-fit and the Compress implant respectively. All cases of stoma hypergranulation and stoma redundant tissue reported on in individuals treated with press-fit or Compress implants occurred in individuals with transfemoral amputation.

Soft tissue complications in individuals with upper extremity amputation were reported in one cohort, with a rate of stoma hypergranulation of 44% in individuals with transhumeral amputation treated with screw implants.[75] No soft tissue complications were reported in individuals with transtibial amputation.

#### Systemic events and death

No cohorts described systemic events such as pulmonary embolism and myocardial infarction and no device-related deaths have been reported.

#### Antibiotics treatment

In four of the 15 cohorts (27%), the use of antibiotics was reported: one in screw implants and three in press-fit implants.[43, 61, 70, 75] Oral antibiotics were used in 26–48% of individuals with transfemoral amputation treated with press-fit implants and in 22% of individuals with transhumeral amputation treated with screw implants. Parenteral antibiotics were used in 1–10% of individuals with transfemoral amputation treated with press-fit implants.

No clear overview of the use of antibiotics for the treatment of infections was provided in the other cohorts.

#### Surgical debridement

The need for surgical debridement was subdivided according to the indication as follows: infection, hypergranulation, stoma redundant tissue or other and was reported in nine of the 15 cohorts (60%), seven of which were cohorts of individuals treated with press-fit implants.[43, 47, 50, 51, 61, 70, 75] The incidence of surgical revision was 11% and 9% in individuals treated with a screw and Compress implant respectively and ranged from 6–77% in individuals treated with press-fit implants. A revision rate of 77%, all due to infection, was reported in the first cohort described by Juhnke et al. [50] consisting of individuals with transfemoral amputation treated with first-generation press-fit implants. The revision rate was 8% in the second cohort after iteration of the surgical technique and implant design, none of which were due to infection. The main overall reasons for surgical revision in all cohorts were stoma redundant tissue and infection.

#### Explantation and re-implantation

The incidence of explantation was described in all cohorts and ranged from 14–19% in individuals treated with a screw implant,[71–75] from 0–57% in individuals treated with a press-fit implant [43, 47, 49, 50, 61, 70] and was 9% in individuals treated with the Compress implant.[51]

Assessment of the level of amputation revealed an explantation rate of 17–18%, 0–13% and 9% in individuals with transfemoral amputation treated with a screw, press-fit or Compress implant, respectively. Two reasons for the explantation of transfemoral implants were intramedullary device breakage, which only occurred in the press-fit implants; and bone fracture, which only occurred in the Compress implant. Implant loosening and infection were other reasons for explantation of transfemoral implants and occurred in both the screw and press-fit implants but not the Compress implant. The rate of explantation was much higher in individuals with transtibial amputation ranging from 42–57%, with Aschoff et al.[47] reporting high rates of implant loosening. All these individuals were treated with press-fit implants. The explantation rate was 17–19% in individuals with transhumeral amputation treated with screw implants. An explantation rate of 14% was reported in the cohort evaluated by Tillander et al.[74] comprising a combination of individuals, all of which being individuals with transfemoral amputation treated with screw implants.

The incidence of re-implantation was reported in 13 of the 15 cohorts (87%); it was performed successfully in 100% of individuals treated with the Compress implant and in 6–40% and 25–100% of the cohorts of individuals treated with screw and press-fit implants, respectively.[43, 47, 49–51, 61, 70, 71, 73–75] Only Tillander et al. [73] reported on re-implantation in individuals with transfemoral amputation treated with screw implants, being succesfull in 6% of individuals all of which explanted due to infection. They did not report on re-implantation rates for the individuals treated with explantation with other etiologies. Thus successful re-implantation rates were unclear in individuals with transfemoral amputation treated with a screw implants while being successful in 50–100% and 100% of individuals with transfemoral amputation treated with a press-fit and Compress implant respectively. Re-implantation was successful in 25% of individuals with transtibial amputation in the cohort described by Juhnke et al.,[49] while the exact rate of successful re-implantation was not clearly reported in the cohort reported by Aschoff et al.[47] Re-implantation was successful in 33% of individuals with transhumeral amputation, and Tillander et al. [74] reported a successful re-implantation rate of 40% in their cohort of individuals with an upper- and lower-extremity amputation treated with screw-fit implants.

#### Peri-prosthetic fracture treatment

The occurrence of peri-prosthetic fracture treatment was described in seven of the 15 cohorts (47%); of which six cohorts involving individuals with transfemoral amputations treated with press-fit implants and one involving individuals treated with the Compress implant.[43, 47, 49–51, 61] In these cohorts, all peri-prosthetic bone fractures were treated surgically and treatment was combined with an implant revision in the cohort of individuals treated with the Compress implant.

## Discussion

This is the first study to provide a complete and detailed overview of device-related complications in both individuals with lower and/or upper extremity amputation treated with screw, press-fit or other types of bone-anchored prostheses, while also providing an overview of complication-related interventions.

The occurrence of explantation of implants was the only outcome reported in all cohorts, followed by re-implantation (87%), infection (73%) and implant loosening (60%). For the purpose of comparison, complications rates reported by Branemark et al. [41], which was excluded due to complete overlap with Tillander et al. [73], that did not come to light in the other cohorts will be included in the discussion (Total infection 67% (grade 1–2: 58%, grade 3: 6%, grade 4: 2%), device fracture: 8% (all of which abutment), implant loosening: 6%, explantation 8%). a) Explantation rates seemed to vary greatly when comparing different implants (screw: 8–19%, press-fit: 0–57%, Compress: 9%), but due to the high explantation rates of transtibial implants (43–57%), all of which were press-fit, these rates provide a biased representation of the outcome. If only explantation rates of transfemoral implants are compared, press-fit implants seem to be less frequently explanted than screw-fit implants (0–13% vs 8–18%) with a similar rate of explantation of the Compress implant (9%), being the only implant that had to be explanted due to a bone fracture. Explantation rates in individuals with transhumeral amputation treated with screw implants ranged from 17–19%. The article by Jonsson et al.[68], which was excluded due to complete overlapping data with Li et al.[71], reported in more detail the explantation rates in individuals with transradial and thumb implants treated with screw implants, being 10% and 30% respectively. b) Re-implantation was typically more successful in individuals treated with a press-fit or Compress implant, especially in individuals with transfemoral amputation (Press-fit: 50–100%, Compress: 100%, screw: 6%); however these rates may also be biased, as only one Compress implant was re-implanted and it is also possible that re-implantation was attempted more often in certain subgroups. The article by Tillander et al.[73] reported on a successful re-implantation rate of 6% in individuals that had their implant explanted due to infection, only they did not report on re-implantation rates of the individuals that underwent explantation on other accounts. c) Total infection rates varied substantially between studies, with no infections occurring in the small cohort treated with the Compress implant and seemingly showing a favorable trend of implant infections (Grade 4) for the screw over the press-fit implant (screw: 2–11%, press-fit: 0–29%); although these numbers, again, are greatly affected by transtibial implants in which there is less expertise. When comparing implant infections between transfemoral screw and press-fit implants (screw: 2–11%, press-fit: 0–3%) there is a considerable difference, and when looking at amputation level (transtibial (press-fit): 29%, upper extremity (screw): 11%) it is clear that there are high rates of implant infections in transtibial implants. d) Again, when examining implant loosening and comparing implants (screw: 3–23%, press-fit: 0–29%, Compress: 0%) a biased representation is created due to the high rate of complications in individuals with a transtibial and upper extremity amputation. When only comparing rates between individuals with a transfemoral amputation, the rates seem to be slightly lower in press-fit implants (screw: 6%, press-fit: 0–3%), with increasing rates in upper extremity screw implants (Thumb: 23%, transhumeral 13%) and very high rates of implant loosening in individuals with press-fit transtibial implants (29%).

Other noteworthy findings concern the incidence of device breakage and surgical revision; a) Device breakages occurred at rates of 0% in the small Compress implant cohort and 8–45% and 0–31% in individuals treated with screw and press fit implants respectively, but were mainly due to breakage of external replaceable parts of the prosthetic system, except for the individuals with transradial implants (27% fixture breakage). Breakage of the intramedullary device was rarely observed in individuals with transfemoral implants, with an incidence of 0% in screw transfemoral implants and 1% in press-fit transfemoral implants. b) The need for surgical revision varies greatly between all cohorts (8–77%), and has only been reported in 60% of cohorts. Infection and stoma redundant tissue appear to be the main reasons for surgical revision, and these rates could be considerably affected by iterations of the implant design and the surgical technique.[50] The treatment of infection with, for instance, antibiotics, and the occurrence of soft tissue complications were greatly under-reported by the included articles, even though multiple articles concluded that infection and soft tissue complications were the most commonly encountered problems in individuals treated with bone-anchored prosthetics.[47, 49, 50, 53]

To help interpret the complication rate of bone-anchored prostheses, a head-on comparison with the complication rates in primary total hip arthroplasty (THA), which is considered standard orthopedic care, with acceptable complication rates has been performed.[76] Gundtoft reported a cumulative 5-year incidence of prosthetic joint infections in 29.077 individuals treated with 32.896 primary THA’s of 1%.[77] These deep infections or prosthetic joint infections are equivalent to the grade 4 infections mentioned above and, especially in the case of press-fit transfemoral bone-anchored implants, show potentially similar results (0–3%). The systematic reviews by van Eck et al. [54], Hebert et al. [55] and Al Muderis et al [56] had an overlapping research question with this review and briefly reported on the complications of bone-anchored prostheses. Of the 12 articles included in this systematic review, only two, [72, 74] six [43, 50, 61, 70, 72, 74] and two [72, 74] were included by van Eck et al., Hebert et al. and Al Muderis et al., respectively, to evaluate complication incidence. The cause of this difference is that we excluded articles with complete overlap and included participants with an upper extremity amputation. It was not possible to compare our result with the above- mentioned reviews because van Eck et al. did not stratify the extracted data, Hebert et al. only presented the data per included article but failed to present overall complication ranges and Al Muderis et al. presented only non-detailed descriptive data.

### Strengths and limitations

A number of factors may have led to distortion of the findings of this review. First, most articles only reported limited complications, with no article providing a complete review of all possibly occurring complications. Explantation was the only complication mentioned in all articles. Second, despite our efforts to prevent overlap, there most likely was partial overlap of patient data in some of the included studies, due to an overlap in the periods of inclusion of individuals (Fig 2); which can lead to duplicate data and may affect outcomes. Third, in many of the included studies, it was unclear how the complications were reported [47, 49, 51, 71], and the study by Tsikandylakis et al. [75] was the only one that reported on the type of examiner that registered complications at follow-up. In most studies, it was unclear whether the complications were collected in specific databases, by investigating electronic patient files or by acquiring information from general practitioners or other hospitals. Fourth, a certain type of selection bias might have occurred, for instance, in the article by Tillander et al. [74], which included individuals attending the clinic for scheduled or emergency visits. Fifth, all included articles were cohort studies, prospective or even retrospective, also giving rise to questions regarding the methodological quality. Sixth, given the small number of individuals included in every study and the varying number of studies reporting certain outcomes, the overall complication rates could be greatly influenced by single outliers. Seventh, the learning curve for the treatment and adaptation of technique and design can also affect complication rates. The article by Juhnke et al. [50] reported a very high incidence of surgical re-intervention in its first cohort, which decreased substantially as a result of iterations of the device design and surgical technique. The article by Hagberg et al. [42], which was excluded due to complete overlap with Tillander et al. [73], also stated that most failures occurred in the early group of individuals that was not treated with a standardized rehabilitation protocol. Eighth, a number of factors may have led to the underestimation of certain complications. It can be suspected that minor complications are likely to be treated by the general practitioner, possibly resulting in an underestimation in the report. Another reason for the possible underestimation of complications is the presence of multiple studies that did not clearly report the occurrence of infections, with some only reporting major complications, such as high grade infections (Grade 3–4), that led to surgical interventions.[47, 49, 50] Complications are often patient-reported, which can also result in an underestimation. Some form of publication bias may have also led to an underestimation of overall complications found in this review, as it is possible that studies with negative outcomes might have not been published. Ninth, it is important to note that conclusions drawn should be interpreted as originating from included studies with a generally weak nature of quality. Assessing the methodological quality of articles reporting complications can lead to difficulties due to the lack of a gold standard classification system to establish complications after bone-anchored prostheses surgery or a consensus regarding specific data collection methods. Other aspects ranked by critical appraisal tools, such as controlling for confounders and the level of blinding, can rarely be avoided because complication data are mostly collected during daily clinical care.

The first and most important strength of this review is that subgroup analyses were performed regarding the implant type and level of amputation, resulting in improved clinical utility. Thus, when more data are available in the future, it might be possible to supply targeted advice regarding the choice of implant type in terms of the level of amputation. We also clarified that, given the way data have been published to date, it is not possible to stratify complications as short- , mid- or long-term complications. More studies with fixed follow-up periods, such as the study by Branemark et al., [41] are necessary to clarify this point. Complications have been well-defined in most studies and regular follow-ups with substantial overlap between different articles, but these follow-ups were not used as specific time points for reporting complications in these publications. A second strong point is that we have given a clear insight in the great amount of patient data overlap through the Gantt chart depicted in Fig 2. To correct for the effect of the overlapping cohorts and duplicate data, we aimed to perform an individual patient data (IPD) meta-analysis. Rather than extracting summary data from the study publications, we searched for the original research data directly from the researchers to exclude any duplicates. Performing this IPD meta-analysis was not possible because the approached researchers were not able to share their original data. A third strong point is the high level of agreement between the two reviewers about ratings of methodological quality.

### Recommendation for future research

As mentioned above, there was no clear consensus in the studies included regarding which complications were reported. In future research, it would be beneficial if all studies would report the same complications in the same manner. A core set should be formulated to provide a representation of the most important complications that should be reported. The content of this core set could be as follows: infection, soft tissue complications, bone fracture, device breakages, implant loosening, explantation, surgical revision, antibiotic use, re-implantation, systemic events and death and uneventful course (Table 4).[43, 50, 61] Within this core set, it would also be beneficial to have strict follow-up times (for example 1, 2, 3, 5 and 10 years). When reporting certain complications, it would be beneficial to follow a certain classification system, such as, for example, the classification system for infection as proposed by Al Muderis et al. Table 1.[61] Furthermore, to interpret the current data in an improved fashion, an IPD meta-analyses is suggested for future research. To facilitate the process of data collection, it is advisable to construct a central database in which all data are stored that follows the core set of above-mentioned complications. We were not able to perform a meta-analysis due to the heterogeneity of the data in terms of outcomes and follow-up intervals. To facilitate a meta-analysis in the future we suggest the following fixed follow-up periods: one, two, five, 10- and 20-year post-operative follow-ups.

## Conclusion

In conclusion, this systematic revealed that in individuals treated with a transfemoral implant the incidence of major complications such as implant infection, implant loosening and explantation was lower in users of a press-fit implant compared to a screw implant. Individuals treated with a transtibial or upper extremity implant and compress implant were underreported, precluding definitive conclusions. The current data revealed that the complication rates encountered in these subgroups of individuals exceed what is deemed acceptable for regular orthopedic interventions. In general, minor complications are most common, such as complications or infections of the soft tissues, which may be greatly affected by the learning curve, implant design and surgical technique, and breakage of external replaceable parts of the implant.

To improve future treatment and research, it will be necessary to formulate a core set of complications that should be reported at fixed time points, as well as to follow a classification system that results in clear and unequivocal data and research. This review could also help professionals and patients in the choice of implant type with respect to the amputation level. However, it should be kept in mind that our conclusions are based on articles of low methodological quality.

## Supporting information

S1 Appendix Search string for each database. (PDF)

S2 Appendix PRISMA checklist. (PDF)
