# Supplementary material for: Complications of bone-anchored prostheses for individuals with an extremity amputation: A systematic review
Source: PLoS One. 2018 Aug 9;13(8):e0201821. doi: 10.1371/journal.pone.0201821 (PMC6084937; doi:10.1371/journal.pone.0201821)
Supplement: S1 Appendix — (PDF) [file pone.0201821.s001.pdf]

Appendix 1: Search string for each database

<u>MEDLINE (accessed via PubMed) => 179 hits:</u>

(Amputation[MeSH] OR Amputees[MeSH] OR Amputation, Traumatic[MeSH] OR Amputation Stumps[MeSH] OR Amput*[Title/Abstract]) AND (osseointegration[MeSH Terms] OR osseointegrat*[Title/Abstract] OR osseo-integrat*[Title/Abstract] OR osseointegrat*[ot] OR bone-anchored prostheses[Title/Abstract] OR boneanchored prostheses[Title/Abstract])

<u>Cochrane Central Register of Controlled Trials => 3 hits:</u>

#1	MeSH descriptor: [Osseointegration] explode all trees
#2	osseointegrat*:ti,ab,kw (Word variations have been searched)
#3	"'osseo-integrated":ti,ab,kw (Word variations have been searched)
#4	"osseo-integrate":ti,ab,kw (Word variations have been searched)
#5	"osseo-integrat":ti,ab,kw (Word variations have been searched)
#6	"osseo-integration":ti,ab,kw (Word variations have been searched)
#7	"bone-anchored prostheses":ti,ab,kw (Word variations have been searched)
#8	"bone-anchored prosthesis":ti,ab,kw (Word variations have been searched)
#9	"bone-anchored prosthetics":ti,ab,kw (Word variations have been searched)
#10	MeSH descriptor: [Amputation] explode all trees
#11	MeSH descriptor: [Amputation Stumps] explode all trees
#12	MeSH descriptor: [Amputation, Traumatic] explode all trees

#13	ampu*:ti,ab,kw (Word variations have been searched)
#14	MeSH descriptor: [Amputees] explode all trees
#15	(#1 or #2 or #3 or #4 or #5 or #6 or #7 or #8 or #9) and (#10 or #11 or #12 or #13 or #14)

EMBASE (accessed via OvidSP) => 206 hits:

(prosthesis implantation.sh. OR osseointegrat*.tw. OR osseo-integrated.tw. OR osseo-integrate.tw. OR osseo-integrat.tw. OR osseo-integration.tw. OR bone-anchored prostheses.tw. OR bone-anchored prosthesis.tw. OR bone-anchored prosthetics.tw.) AND (amputation.sh. OR amput*.tw. OR disabled person.sh. OR disabled person.tw.

CINAHL => 60 hits:

((MH "Amputation+") OR (MH "Amputation, Traumatic") OR (MH "Amputees") OR (TI amput*) OR (AB amput*)) AND ((TI osseointegrat*) OR (AB osseointegrat*) OR (TI "osseo-integrat*") OR (AB "osseo-integrat*") OR (TI "bone-anchored prosthe*") OR (AB "bone-anchored prosthe*"))

Web of Science => 177 hits:

TS=((osseointegrat* OR osseo-integrat* OR bone-anchored prosthe*) AND (amput*))

System for information on Grey Literature (Open Grey) => 1 hit

(osseointegrat* OR "osseo-integrated" OR "osseo-integration" OR "bone-anchored prostheses" OR "bone-anchored prosthesis") AND (amput*)

Total: 626 hits
